# Rural–urban differences in dental opioid prescribing among adolescent/young adult and adult Medicaid beneficiaries

**DOI:** 10.3389/fpubh.2024.1465206

**Published:** 2024-10-17

**Authors:** Carla Shoff, Alex Sheen, Luping Qu, Natalia I. Chalmers

**Affiliations:** ^1^Office of the Administrator, Centers for Medicare & Medicaid Services, Baltimore, MD, United States; ^2^Department of Pediatric Dentistry, New York University College of Dentistry, New York, NY, United States

**Keywords:** dental, opioid, prescribing, oral health, prescription, rural, urban, Medicaid

## Abstract

**Introduction:**

There are ongoing concerns about opioid prescribing for surgical and non-surgical dental needs among adolescent/young adult and adult patients. Although there are known differences in the overall opioid prescription rates in rural areas compared to urban areas, the contribution of dental opioid prescriptions is still unclear. This study aims to examine the factors associated with receiving an opioid prescription following a dental visit.

**Materials and methods:**

This cross-sectional study utilized the 2021 Centers for Medicare & Medicaid Services unredacted Transformed Medicaid Statistical Information System Analytic Files to examine Medicaid and CHIP adolescent/young adult beneficiaries aged 12–20 and adults aged 21–64 who are non-dually eligible for Medicare and had a dental visit in 2021. Multilevel logistic regression models were used to predict the odds of receiving a dental opioid prescription.

**Results:**

The results of the adolescent/young adult models show that for every percentage point increase in the percentage of non-Hispanic Black residents in a county, the odds of receiving a dental opioid prescription increase by 0.8% in rural areas. However, with every percentage point increase in the Hispanic population, the odds of receiving a dental opioid prescription decrease by 0.3% in rural areas and 0.7% in urban areas. The adult models show that compared to non-Hispanic white beneficiaries, non-Hispanic Black beneficiaries are 8% more likely to receive a dental opioid prescription if they live in rural areas and 18% more likely if they live in urban areas, while all other racial and ethnic groups are significantly less likely to receive a dental opioid prescription. With every unit increase in the concentrated disadvantage index, the odds of receiving a dental opioid prescription increase by 17% among rural adults and 24% among urban adults.

**Discussion:**

Our findings on rural–urban disparities in opioid prescriptions suggest that prescription patterns in dental settings are significant and inequitable across various beneficiary- and county-level factors and areas of residence. These variations in prescription patterns highlight the known disparities in access to preventive dental care and the need for targeted interventions to address the healthcare needs of rural residents.

## Introduction

The overall rate of opioid prescribing in the United States (US) has steadily decreased since its peak in 2012 (1). Several initiatives, including prescription drug monitoring programs (PDMPs), clinical guidelines, and state opioid prescribing limits, have helped contribute to the overall decline in opioid prescriptions (2–5). Despite these efforts, opioid prescriptions remain elevated for specific populations and geographical areas, with prescription practices varying widely by practitioner specialty (6–9). While dentists typically prescribe opioids for analgesic purposes related to dental pain or after dental procedures such as tooth extraction or surgery, research and current guidelines now reflect that nonsteroidal anti-inflammatory drugs (NSAIDs) are more effective in a majority of cases (10, 11). Nevertheless, dentists remain one of the nation’s highest-prescribing healthcare specialties (12–14), second only to internal medicine (16.4%) (6). In particular, adolescent and young adult populations are significant recipients of dental opioid prescriptions (15). In 2009, dentists accounted for 31% of opioid prescriptions to children and adolescents aged 10 through 19 years (16). Moreover, from 2019 to 2015, the largest increase in dental opioid prescriptions was seen among 11 through 18 years olds (12). The effects of this are significant. Multiple studies have found that adolescents and young adults are often first exposed to opioids through dental providers and at increased risk for future opioid misuse and abuse (15, 17). Another study showed that adolescents and young adults receiving opioid prescriptions from dentists were at increased risk for subsequent substance-related morbidity (18). Additionally, studies have shown that dental-related prescriptions are often not fully used, which may increase the risk of opioid diversion and misuse of the leftover pills (19, 20).

Previous studies have primarily found higher overall opioid prescription rates in rural areas (21–23), but studies investigating dental opioid prescriptions have found no rural/urban differences (24, 25). However, these studies only examined oral maxillofacial surgeons (24, 25). Rural areas have been subject to many oral health challenges, with previous reports documenting higher rates of edentulism and poorer self-reported oral health scores for children (26, 27). In addition, there are several structural challenges that rural areas face, such as less access to dental care, higher rates of poverty, transportation difficulties, and lack of dental insurance (28, 29). Studies have identified similar structural challenges as reasons for higher opioid prescriptions and misuse in rural areas (30, 31).

Previous studies have explored provider-, procedure-, and beneficiary-specific factors associated with increased dental-related opioid prescriptions (15, 32–39). Studies examining beneficiary-level factors have found that higher opioid prescriptions differed by a wide variety of predictors. One study identified higher rates of opioid prescriptions among patients who were low-income, less educated, and insured by Medicaid (39). Other studies have reported that patients who are ‘young,’ ‘female,’ or ‘Non-Hispanic (NH) Black’ receive opioid prescriptions in dental settings at higher rates (15, 36–39). Further, several studies have investigated the complex interplay between beneficiary- and county-level factors within urban and rural settings and their associations with general opioid prescription rates (30, 31, 40, 41). However, to our knowledge, no studies examine the association between patient demographic factors, county characteristics, and dental opioid prescriptions. In addition, no studies explore how the association between patient demographic factors, county characteristics, and dental opioid prescriptions vary across urban and rural settings. This study aims to address these gaps in the literature by examining the factors associated with receiving a dental opioid prescription, considering both beneficiary and county characteristics. These characteristics will be examined separately for adolescents and adults, and how these characteristics differ across rural and urban areas will be considered.

## Materials and methods

### Data sources

This study utilized multiple data sources, including the 2021 Centers for Medicare & Medicaid Services (CMS) unredacted Transformed Medicaid Statistical Information System (T-MSIS) Analytic Files (TAF) Research Identifiable Files (RIF). Specifically, the Demographic and Eligibility, Other Services, and Pharmacy files were utilized and were accessed through the CMS Chronic Conditions Data Warehouse (CCW) (42). The American Dental Association (ADA) Dentist Data 2022 files were used to identify dental providers (43). The Economic Research Service Rural–Urban Commuting Area Codes were used to determine the rural/urban status of the beneficiary’s residence (44). This data was linked to the T-MSIS data using the beneficiary’s ZIP code. The US Census Bureau’s American Community Survey (ACS) 5-year (2008–2021) estimates were used to calculate the county characteristics measures (45). This data was linked to the T-MSIS data using the beneficiary’s Federal Information Processing Standard (FIPS) code. The study was covered by the Common Rule exemption, 45 CFR 46.104(d)(4)(iv), and did not require institutional review board review.

### Cohort design and population

This study includes Medicaid and CHIP adolescent/young adult beneficiaries aged 12 to 20 and adults aged 21 to 64. All beneficiaries included in this study are non-dually eligible for Medicare. Beneficiaries must have had a dental visit in 2021. Beneficiaries are considered to have had a dental visit if they have a billed claim with any Code on Dental Procedures and Nomenclature (CDT code) for a dental procedure.

To be included in this study, beneficiaries must live in a state that does not need to be excluded for data quality concerns, according to the CMS DQ Atlas (46). States assigned as high concern or unusable on the following topics were excluded from all analyses: professional services claims volume, professional services procedure codes, prescription claims volume, prescription National Drug Codes (NDC), and linking claims to beneficiaries, resulting in beneficiaries from 9 states excluded from all analyses [Arkansas (AR), Florida (FL), Massachusetts (MA), Maine (ME), Minnesota (MN), Mississippi (MS), North Carolina (NC), New Jersey (NJ), and Utah (UT)] (46). Beneficiaries from 14 states are excluded due to race and ethnicity data quality concerns, 2 (MA and UT) of which were already excluded for claims data quality concerns [Arizona (AZ), Connecticut (CT), District of Columbia (DC), Hawaii (HI), Iowa (IA), Louisiana (LA), MA, New York (NY), Oregon (OR), Rhode Island (RI), South Carolina (SC), Tennessee (TN), UT, Wyoming (WY)] (46). Combining the claims data and race and ethnicity data quality exclusions, 21 states (AR, AZ, CT, DC, FL, HI, IA, LA, MA, ME, MN, MS, NC, NJ, NY, OR, RI, SC, TN, UT, WY) were excluded from all analyses stratified by race and ethnicity. After exclusions, 6,419,051 adolescent/young adult beneficiaries and 5,583,051 adult beneficiaries are included in the analyses that were not stratified by race and ethnicity, and a subset of 4,938,928 adolescent/young adult beneficiaries and 3,977,143 adult beneficiaries when stratified by race and ethnicity.

### Outcome, demographic, and residential covariates

The outcome variable for this study is a dichotomous measure of whether a beneficiary received a dental opioid prescription. Opioid prescriptions were identified using NDC for prescriptions filled and paid by Medicaid, which are included in the pharmacy claims file. To be considered a dental opioid prescription, two requirements had to be met. First, the opioid had to be prescribed by a dental provider (a dental provider is defined as a dentist according to the ADA dentist data file (43) or as a provider with a dental taxonomy code according to the National Uniform Claim Committee’s (NUCC) Health Care Provider Taxonomy Code Set) (47). Second, the opioid prescription must have been filled within 7 days of a dental visit. To ensure that the opioid prescription is truly from a dental visit, all the beneficiary’s medical claims were also reviewed. If a provider billed for any medical visit or procedure and an opioid was prescribed within 7 days, the opioid prescription was excluded from the analysis. The reason for the exclusion is so that we can be extra confident that the opioid prescribed within 7 days of a dental visit did not come from a medical visit, which was also within 7 days of the dental visit.

This study includes beneficiary characteristics, including age, sex, race and ethnicity, as well as county characteristics, including rural/urban status, percentage of NH Black population, percentage of Hispanic population, concentrated disadvantage, and residential stability. Age is treated as a categorical variable using age 12–14 (reference group), age 15–17, and age 18–20. Sex is a categorical variable: male (reference group) and female. Race and ethnicity is treated as a categorical variable with the following racial/ethnic groups: American Indian/Alaskan Native (AI/AN), Asian/Pacific Islander (A/PI), Non-Hispanic (hereafter, NH) Black, Hispanic, NH White (reference group), and multiracial/other race/unknown race. Beneficiaries with missing race/ethnicity data are included in the multiracial/other race/unknown category. The percentage of NH Black and Hispanic population are continuous variables. Concentrated disadvantage is a continuous index variable calculated by applying principal components analysis to 5 variables from the ACS: logged median family income, unemployment rate, percentage of families headed by women, and percentage of the population receiving public assistance. All factor loadings are higher than 0.55, and this single factor accounts for 60 percent of the variance. Higher values suggest a higher concentrated disadvantage within the county. Residential stability is a continuous variable that is the average of two standardized indicators: the percentage of owner-occupied housing units and the percentage of households living in the same housing unit for at least 5 years. These county-level variables have been used in previous studies on opioid prescribing and opioid use disorder (41, 48–53).

### Statistical analysis

The study population counts and rates per 1,000 Medicaid/CHIP beneficiaries who had a dental visit, as well as the opioid dental prescribing rate per 1,000 Medicaid/CHIP beneficiaries who had a dental visit, are presented for overall and by rural and urban status. Chi-square tests were used to test for significant differences in the rates across categories within each group, and whether the category-specific rates significantly differ across rural and urban areas. Multilevel logistic regression models were used to predict the odds of receiving a dental opioid prescription. The models include beneficiary characteristics in level-one and county characteristics in level-two. The county FIPS code is included in the models as a level-two random intercepts parameter to adjust for the similarity of beneficiaries residing within the same county. Separate multilevel logistic regression models were estimated for rural and urban beneficiaries. Wald tests were implemented to test whether the magnitude of the effects significantly differ across the rural and urban models. This approach is the equivalent of a full interaction model. Statistical significance was set at *p* ≤ 0.05; all *p* values were 2-tailed. Results are presented as adjusted odds ratios (aORs) and corresponding 95% confidence intervals (CI). Multicollinearity was assessed with variation inflation factors (VIF) and was identified in the models because no VIF were above 2. Analyses were conducted with SAS Enterprise Guide 7.1 and Stata 18.0 (54, 55). Maps were created with ESRI ArcGIS Pro 3.3.1 (56).

## Results

### Characteristics of study population and prevalence of opioid dental prescribing: adolescent/young adults

There are 6,419,051 adolescent/young adult beneficiaries in this study; of these, 2,756,216 (429 per 1,000) are 12–14 years old, followed by 2,291,730 (357 per 1,000) 15 to 17 years old (Table 1). There are more females (516 per 1,000) than males. Approximately one-third of adolescents and young adults are Hispanic (374 per 1,000), and another one-third are NH white (332 per 1,000).

**Table 1:** Study population characteristics and opioid dental prescribing rates per 1,000 Medicaid/CHIP beneficiaries who had a dental visit, 2021.

	Study population			Opioid dental prescribing			
	Overall	Rural	Urban	Overall	Rural	Urban	
	No. (rate)^a^	No. (rate)^a^	No. (rate)^a^	No. (rate)^a^	No. (rate)^a^	No. (rate)^a^	
	Adolescents/Young Adults (*N* = 6,420,460)						*p*-value
Overall	6,419,051 (1000.00)	1,083,269 (168.76)	5,335,782 (831.24)	225,757 (35.17)	51,453 (47.50)	174,304 (32.67)	≤0.001
Age group							≤0.001
Age 12–14	2,756,216 (429.38)	470,883 (434.69)	2,285,333 (428.24)	17,351 (6.30)	4,280 (9.09)	13,071 (5.72)	≤0.001
Age 15–17	2,291,730 (357.02)	390,667 (360.64)	1,901,063 (356.24)	94,685 (41.32)	22,105 (56.58)	72,580 (38.18)	≤0.001
Age 18–20	1,371,105 (213.60)	221,719 (204.68)	1,149,386 (215.38)	113,721 (82.94)	25,068 (113.06)	88,653 (77.13)	≤0.001
Sex							≤0.001
Female	3,314,333 (516.33)	564,598 (521.20)	2,749,735 (515.27)	129,165 (38.97)	29,955 (53.06)	99,210 (36.08)	≤0.001
Male	3,104,718 (483.67)	518,671 (478.80)	2,586,047 (484.59)	96,592 (31.11)	21,498 (41.45)	75,094 (29.04)	≤0.001
Race and ethnicity							≤0.001
American Indian/Alaskan Native	62,112 (12.58)	31,678 (37.99)	30,434 (7.41)	3,557 (57.27)	1,746 (55.12)	1,811 (59.51)	0.019
Asian/Pacific Islander	189,503 (38.37)	6,507 (7.80)	182,996 (44.58)	5,570 (29.39)	303 (46.57)	5,267 (28.78)	≤0.001
Non-Hispanic Black	831,052 (168.27)	63,574 (76.24)	767,478 (186.96)	28,071 (33.78)	3,529 (55.51)	24,542 (31.98)	≤0.001
Hispanic	1,849,278 (374.43)	184,217 (220.91)	1,665,061 (405.61)	48,896 (26.44)	6,544 (35.52)	42,352 (25.44)	≤0.001
Non-Hispanic White	1,639,975 (332.05)	482,013 (578.02)	1,157,962 (282.08)	70,053 (42.72)	23,480 (48.71)	46,573 (40.22)	≤0.001
Multiracial/Other Race/Unknown	367,008 (74.31)	65,910 (79.04)	301,098 (73.35)	16,337 (44.51)	3,766 (57.14)	12,571 (41.75)	≤0.001

	Adults (*N* = 5,583,976)						
Overall	5,583,051 (1000.00)	844,277 (151.22)	4,738,774 (848.78)	524,619 (93.97)	102,113 (120.95)	422,506 (89.16)	≤0.001
Age group							≤0.001
Age 21–34	2,471,296 (442.64)	378,679 (448.52)	2,092,617 (441.59)	250,298 (101.28)	49,527 (130.79)	200,771 (95.94)	≤0.001
Age 35–49	1,770,904 (317.19)	276,432 (327.42)	1,494,472 (315.37)	167,111 (94.36)	33,094 (119.72)	134,017 (89.68)	≤0.001
Age 50–64	1,340,851 (240.16)	189,166 (224.06)	1,151,685 (243.03)	107,210 (79.96)	19,492 (103.04)	87,718 (76.16)	≤0.001
Sex							≤0.001
Female	3,606,991 (646.06)	562,227 (665.93)	3,044,764 (642.52)	339,910 (94.24)	66,825 (118.86)	273,085 (89.69)	≤0.001
Male	1,976,060 (353.94)	282,050 (334.07)	1,694,010 (357.48)	184,709 (93.47)	35,288 (125.11)	149,421 (88.21)	≤0.001
Race and ethnicity							≤0.001
American Indian/Alaskan Native	60,374 (15.18)	26,602 (40.59)	33,772 (10.17)	6,177 (102.31)	2,403 (90.33)	3,774 (111.75)	≤0.001
Asian/Pacific Islander	245,473 (61.72)	5,359 (8.18)	240,114 (72.29)	12,558 (51.16)	434 (80.99)	12,124 (50.49)	≤0.001
Non-Hispanic Black	766,792 (192.80)	31,941 (48.73)	734,851 (221.23)	91,470 (119.29)	5,590 (175.01)	85,880 (116.87)	≤0.001
Hispanic	849,395 (213.57)	69,407 (105.89)	779,988 (234.82)	65,410 (77.01)	6,555 (94.44)	58,855 (75.46)	≤0.001
Non-Hispanic White	1,760,358 (442.62)	479,386 (731.40)	1,280,972 (385.64)	192,930 (109.60)	57,481 (119.91)	135,449 (105.74)	≤0.001
Multiracial/Other Race/Unknown	294,751 (74.11)	42,744 (65.21)	252,007 (75.87)	27,649 (93.80)	5,323 (124.53)	22,326 (88.59)	≤0.001

^a^Rates per 1,000 beneficiaries.

**Figure 1:**
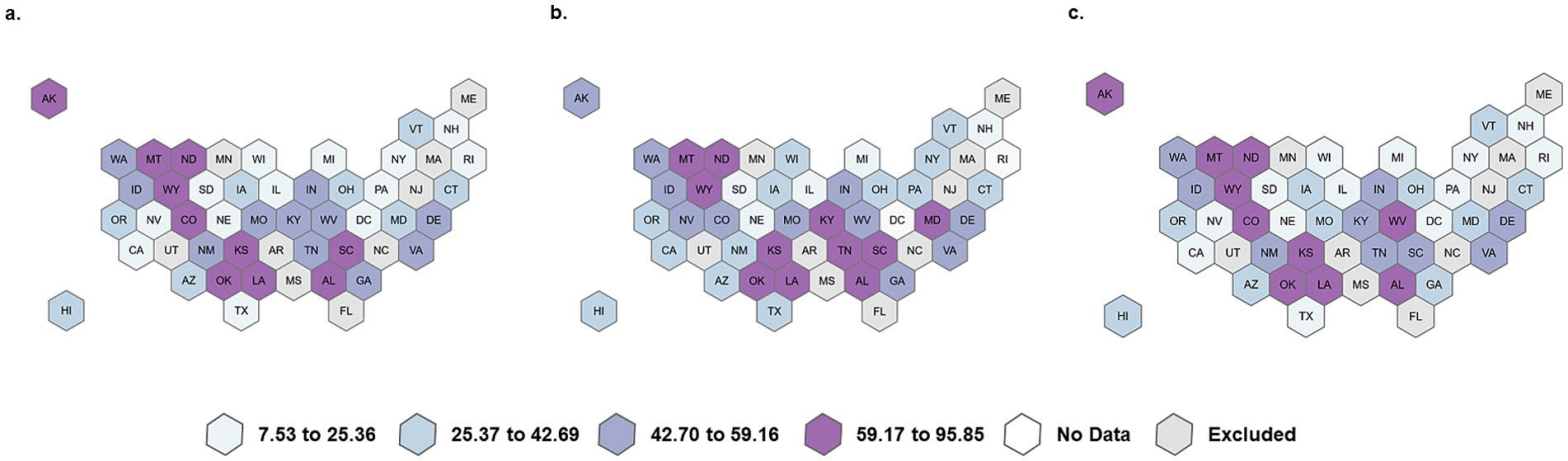
Overall, rural, and urban dental opioid prescribing rate per 1,000 adolescent/young adult Medicaid/CHIP beneficiaries. **(a)** Overall rate per 1,000, **(b)** Rural rate per 1,000, and **(c)** Urban rate per 1,000.

Figures 1a–c display the rates of receiving a dental opioid prescription per 1,000 adolescent/young adult beneficiaries by state. The maps display the overall rate (Figure 1a), the rural rate (Figure 1b), and the urban rate (Figure 1c). The rates vary across states, with the lowest rate being 8 beneficiaries per 1,000 and the highest 96 beneficiaries per 1,000. The state-level rural rates are higher than the overall rates and urban rates.

Table 1 shows the rates of opioid dental prescribing by age group, sex, and race and ethnicity, overall and by rural and urban status. The rates are significantly higher among older age groups (*p* ≤ 0.001). Among 12 to 14-year-olds, 6 beneficiaries per 1,000 received a dental opioid prescription compared to 41 beneficiaries per 1,000 for 15 to 17-year-olds and 83 per 1,000 for 18 to 20-year-olds. Among all age groups, the rates are significantly higher in rural areas compared to urban areas (*p* ≤ 0.001). For example, among beneficiaries 18 to 20 years of age, the rate is 113 per 1,000 in rural areas compared to 77 per 1,000 in urban areas.

Adolescent/young adult female beneficiaries have significantly higher dental opioid prescription rates than male beneficiaries, and these rates are significantly higher in rural areas than they are in urban areas (*p* ≤ 0.001). Among female beneficiaries living in rural areas, the rate is 53 per 1,000 compared to 36 per 1,000 for their urban counterparts. Among male beneficiaries, the rate is 41 per 1,000 in rural areas and 29 per 1,000 in urban areas.

The rates have statistically significant differences across all racial and ethnic groups. Looking at the overall rates, AI/AN beneficiaries have the highest rates at 57 beneficiaries per 1,000, followed by multiracial and other race beneficiaries at 45 per 1,000 and NH white beneficiaries at 43 per 1,000. Rural beneficiaries have higher rates than urban beneficiaries for every racial/ethnic group, except for AI/AN beneficiaries. In rural areas, the rates are highest among multiracial and other race beneficiaries at 57 per 1,000, followed by NH Black and AI/AN beneficiaries at 55 per 1,000. In urban areas, the rates are highest among AI/AN beneficiaries (60 per 1,000), followed by multiracial and other race beneficiaries (42 per 1,000) and NH white beneficiaries (40 per 1,000).

### Characteristics of study population and prevalence of opioid dental prescribing: adults

**Figure 2:**
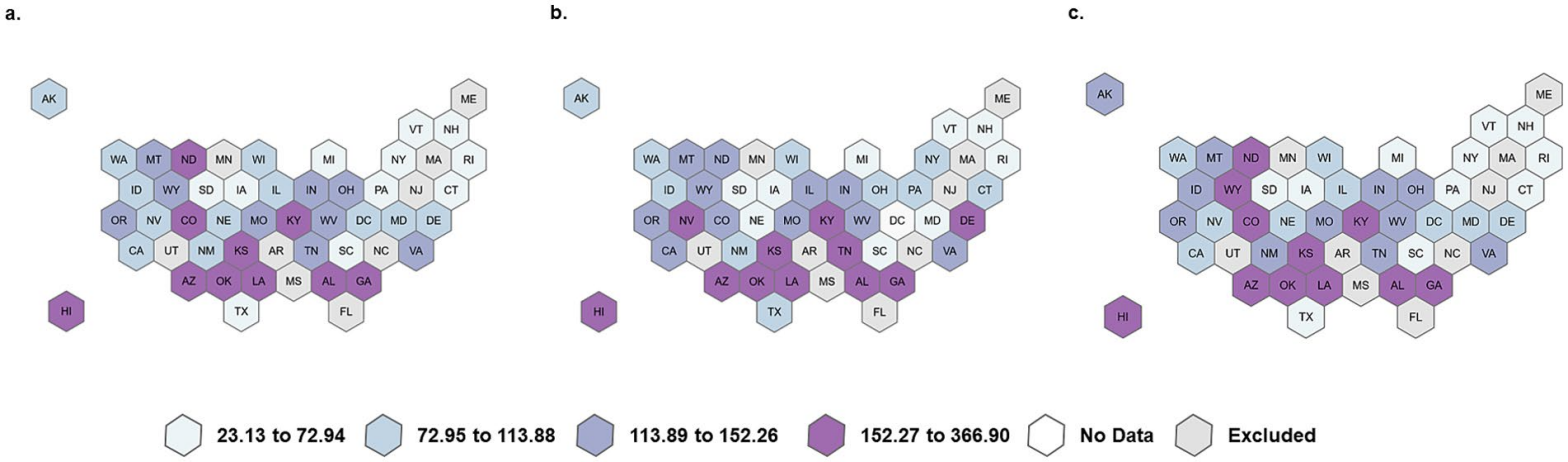
Overall, rural, and urban dental opioid prescribing rate per 1,000 adult Medicaid beneficiaries. **(a)** Overall rate per 1,000, **(b)** Rural rate per 1,000, **(c)** Urban rate per 1,000.

There are 5,583,051 adult beneficiaries in this study; of these, 2,471,296 (443 per 1,000) are 21–34 years old, followed by 1,770,904 (317 per 1,000) 35–49 years old (Table 1). There are more females (646 per 1,000) than males. Almost half of the adults are NH white (443 per 1,000), followed by Hispanic (214 per 1,000) and NH Black (193 per 1,000). The dental opioid prescribing rates among adults are shown in Figures 2a–c. Among adult beneficiaries, the state-level rates vary from 23 per 1,000 to 347 per 1,000. States with the highest rates are generally located in the South and Southwest.

Among adult beneficiaries, the rates significantly decrease with each older age group. Beneficiaries aged 21 to 34 who live in rural areas have the highest dental opioid prescription rates at 131 per 1,000 beneficiaries. Among adults in general and adults living in urban areas, the rates of receiving a dental opioid prescription are higher for females than males. However, among adults living in rural areas, the rates of receiving a dental opioid prescription are significantly higher for males (125 per 1,000) than they are for females (119 per 1,000).

While AI/AN beneficiaries were the racial/ethnic group with the highest overall rates of dental opioid prescriptions for adolescent/young adult beneficiaries, among adults, NH Black beneficiaries have the highest rates (119 per 1,000). As was the case for adolescent/young adult beneficiaries, adult beneficiaries have higher rates in rural areas than in urban areas for every racial and ethnic group, except for AI/AN beneficiaries. In rural areas, the rates are highest among NH Black beneficiaries (175 per 1,000), followed by multiracial and other race beneficiaries at 125 per 1,000, and NH white beneficiaries at 120 per 1,000. In urban areas, the rates are highest among NH Black beneficiaries (117 per 1,000), followed by AI/AN beneficiaries at 112 per 1,000 and NH White beneficiaries (106 per 1,000).

### Characteristics associated with receiving a dental opioid prescription: adolescent/young adults

The multilevel logistic regression model results are reported in Table 2. As shown in the adolescent/young adult models, compared to beneficiaries aged 12–14, beneficiaries aged 15–17 are 6.80 times (aOR, 6.80; 95% CI, 6.55–7.06) more likely to receive a dental opioid prescription if they live in a rural area and 7.22 times (aOr, 7.22; 95% CI, 7.06–7.37) more likely if they live in an urban area. Beneficiaries 18 to 20 years old are 14.59 times (aOR, 14.59; CI, 14.04–15.16) more likely to receive a dental opioid prescription compared to beneficiaries aged 12–14 if they live in a rural area and 15.95 times (aOR, 15.95; CI, 15.61–16.29) more likely if they live in an urban area. The odds of receiving a dental opioid prescription are 19% higher for females than they are for males among beneficiaries living in rural areas and 16% higher for females compared to males among beneficiaries living in urban areas. Among rural adolescent/young adult beneficiaries, the odds of receiving a dental opioid prescription are 21% lower for Hispanic beneficiaries compared to NH white beneficiaries. Among urban beneficiaries, when compared to NH white beneficiaries, all racial and ethnic groups are significantly less likely to receive a dental opioid prescription, except for AI/AN beneficiaries whose odds of receiving a dental opioid prescription are not significantly different from NH whites.

**Table 2:** Multilevel logistic regression model predicting the odds of receiving a dental opioid prescription among Medicaid/CHIP beneficiaries who had a dental visit, 2021.

	Adolescents/Young adults		
	Rural (*N* = 828,906)	Urban (*N* = 4,089,586)	Wald test
	aOR (95% CI)^a^	aOR (95% CI)^a^	*p*-value
Age group (Ref: Age 12–14)			
Age 15–17	6.800 (6.545–7.064)b	7.216 (7.061–7.373)b	0.008
Age 18–20	14.590 (14.044–15.157)b	15.945 (15.607–16.291)b	0.000
Sex (Ref: Male)			
Female	1.187 (1.162–1.212)b	1.159 (1.146–1.173)b	0.063
Race and Ethnicity (Ref: Non-Hispanic White)			
American Indian/Alaskan Native	1.054 (0.988–1.125)	1.002 (0.947–1.059)	0.338
Asian/Pacific Islander	0.940 (0.833–1.061)	0.843 (0.817–0.869)b	0.063
Non-Hispanic Black	0.966 (0.924–1.010)	0.891 (0.875–0.907)b	0.001
Hispanic	0.794 (0.765–0.824)b	0.813 (0.799–0.827)b	0.184
Multiracial/Other Race/Unknown	0.962 (0.925–1.001)	0.912 (0.893–0.933)b	0.022
County characteristics			
Percent Non-Hispanic Black	1.008 (1.004–1.012)b	1.003 (1.000–1.007)	0.299
Percent Hispanic	0.997 (0.995–1.000)b	0.993 (0.990–0.996)b	0.114
Social Disadvantage Index	1.088 (1.031–1.147)b	1.147 (1.074–1.226)b	0.783
Residential Stability	0.921 (0.878–0.965)b	0.988 (0.941–1.037)	0.000

	Adults		
	Rural (*N* = 651,010)	Urban (*N* = 3,305,726)	Wald test
	aOR (95% CI)^a^	aOR (95% CI)^a^	*p*-value
Age group (Ref: Age 21–34)			
Age 35–49	0.895 (0.880–0.911)b	0.936 (0.928–0.945)b	0.000
Age 50–64	0.758 (0.743–0.774)b	0.849 (0.841–0.857)b	0.000
Sex (Ref: Male)			
Female	0.884 (0.869–0.898)b	0.950 (0.943–0.958)b	0.000
Race and Ethnicity (Ref: Non-Hispanic White)			
American Indian/Alaskan Native	0.901 (0.852–0.952)b	0.929 (0.896–0.964)b	0.093
Asian/Pacific Islander	0.635 (0.574–0.703)b	0.542 (0.532–0.554)b	0.001
Non-Hispanic Black	1.077 (1.039–1.117)b	1.177 (1.165–1.189)b	0.000
Hispanic	0.782 (0.755–0.809)b	0.782 (0.773–0.792)b	0.006
Multiracial/Other Race/Unknown	0.866 (0.839–0.894)b	0.797 (0.785–0.809)b	0.000
County characteristics			
Percent Non-Hispanic Black	1.018 (1.014–1.023)b	1.009 (1.005–1.012)b	0.000
Percent Hispanic	0.996 (0.993–0.999)b	0.991 (0.988–0.994)b	0.000
Concentrated Disadvantage	1.173 (1.106–1.244)b	1.243 (1.172–1.318)b	0.000
Residential Stability	0.926 (0.879–0.975)b	0.965 (0.922–1.009)	0.301

aOR, adjusted odds ratio; CI, confidence interval. ^a^Models adjusted for all variables in the table, and all models were clustered at the county-level. ^b^*p* ≤ 0.05.

As for the county characteristics, for every percentage point increase in the percentage of NH Black residents, the odds of receiving a dental opioid prescription increase by 0.8% in rural areas. However, with every percentage point increase in the Hispanic population, the odds of receiving a dental opioid prescription decrease by 0.3% in rural areas and 0.7% in urban areas. With every unit increase in the concentrated disadvantage index, the odds of receiving a dental opioid prescription increase by 9% in rural areas and 15% in urban areas. In rural areas, adolescents and young adults who live in counties with more residential stability have 8% lower odds of receiving a dental opioid prescription.

### Characteristics associated with receiving a dental opioid prescription: adults

As shown in the adult rural and urban models, compared to adults aged 21 to 34, older beneficiaries are significantly less likely to receive a dental opioid prescription. Adult female beneficiaries are significantly less likely to receive a dental opioid prescription compared to males, and the odds are significantly lower for females living in rural areas (12%) than in urban areas (5%). The relationship between race and ethnicity and receiving a dental opioid prescription was different for adults than it was for adolescents and young adults. Among adults, compared to NH white beneficiaries, all racial and ethnic groups are significantly less likely to receive a dental opioid prescription except for NH Black beneficiaries, who are 8% more likely to receive a dental opioid prescription if they live in rural areas and 18% more likely if they live in urban areas.

The county characteristics results show similar patterns for adolescent/young adult beneficiaries. However, the effect of concentrated disadvantage is much stronger for adult beneficiaries. With every unit increase in the concentrated disadvantage index, the odds of receiving a dental opioid prescription increase by 17% among rural adults and 24% among urban adults.

## Discussion

This study revealed that the relationship between age and the rate of beneficiaries receiving a dental opioid prescription is an inverted U-shape. The rates among adolescent/young adult beneficiaries increased with each age group, and among adult beneficiaries, the rates decreased with each age group. We also discover that racial disparities are age-dependent, with AI/AN adolescents/young adults and NH Black adults having the highest overall dental opioid prescription rates. Previous studies have primarily reported that patients who are NH white or NH Black have the highest opioid prescription rates (15, 36–39). Still, these studies did not analyze racial disparities by age group or include AI/AN beneficiaries in the analyses.

The finding that AI/AN adolescent/young adult beneficiaries have the highest dental opioid prescription rates highlights the alarming trend around the over-prescribing of opioids to AI/AN children that should be further explored. Almost half of AI/AN children live in dental care shortage areas, which can hinder access to regular and routine dental visits. Without periodic visits and early intervention, the progression of the disease can eventually lead to severe dental pain. If treatment can be rendered, follow-up appointments may still be challenging. While guidelines have demonstrated that NSAIDs are equally effective in managing dental pain, dental providers may still choose to prescribe opioids as a precautionary measure (57–59). Future investigations into dental opioid prescribing in AI/AN populations can better inform prescribing policies. They may also provide more insight into needed changes in the oral health care delivery system for AI/AN patients to decrease the need for analgesic prescriptions overall. Despite having the highest opioid prescription rates among adolescents/young adults, we did not find that AI/AN adolescent/young adults were significantly more likely to receive an opioid than their NH white counterparts, which previous studies for both general and dental opioid prescriptions concluded as well (60, 61).

Previous studies have primarily shown that overall opioid prescription rates are higher in rural areas (21–23). Our findings reveal higher dental opioid prescription rates in rural areas as well as provide additional perspective by demonstrating that the likelihood of receiving a dental opioid prescription in an urban vs. rural setting can significantly vary depending on beneficiary-level factors such as sex, age, and race and ethnicity. For example, among adolescents/young adults, the likelihood of receiving a dental opioid prescription was significantly lower among NH Black and multiracial/other race beneficiaries in urban areas than it was in rural areas. Among adults, the likelihood of receiving a dental opioid prescription was significantly higher among NH Black urban adults than it was for NH Black rural adults. It is beyond the scope of our study to explore causative drivers for these differences. Still, our findings confirm that the rurality or urbanicity of residence cannot solely predict the odds of receiving a dental opioid prescription and that beneficiary-level characteristics also need to be considered. These findings suggest that successfully addressing disparities in dental opioid prescribing must be sensitive to both individuals and their surrounding environment (62).

County-level predictors showed consistent effects across both adolescents/young adults and adults, but opposite effects were observed between areas with a higher percentage of NH Black residents (higher odds) and a higher percentage of Hispanic residents (lower odds); this finding suggests that race and ethnicity are not only significant at a beneficiary-level but also at a population level. Several studies have attributed racial disparities in opioid prescription patterns to factors such as providers’ conscious and unconscious racial biases as well as systemic structural racism such as segregated neighborhoods and lower density of healthcare providers (36, 63, 64). Our findings may reflect the broader impact these collective biases and disparities can have on entire communities’ dental and medical services access. When considering the impact of county-level factors, we found that residential stability is protective in rural areas for both adolescents/young adults and adults. Studies have shown that residential stability is more common in rural settings and has better health outcomes mediated by communal benefits such as greater social cohesion and community support (65–67). Our findings suggest that these social benefits may also facilitate a lower likelihood of receiving a dental opioid prescription. Yang et al. hypothesized that increased residential stability may not only decrease patient demand for opioids but may also disincentivize providers from prescribing opioids in these communities due to stronger communal ties (41). Future studies that further identify and investigate unique urban and rural characteristics that influence opioid prescribing may help better guide local and community-based initiatives seeking to curb the over-prescribing of opioids in dental settings.

### Limitations

Our study has some noted limitations. First, our findings only account for Medicaid/CHIP beneficiaries and may not be generalizable to the US population. Future studies may investigate these disparities in the commercially insured and uninsured populations. Second, although our primary measure focused on whether a beneficiary was prescribed opioids, we did not consider other important aspects of opioid prescribing, such as the morphine milligram equivalents and the number of days supplied. Third, we are limited to the variables included in Medicaid administrative claims data, which could result in omitted variable bias.

## Conclusion

Our findings on rural–urban disparities in dental opioid prescribing suggest that prescription patterns in dental settings are significant and inequitable across various beneficiary- and county-level factors as well as residence. These variations in prescription patterns indicate that there is not one solution that can address the disparities in opioid prescribing; in fact, developing interventions and policies that have identified targeted factors that account for the local populations and community structure may provide a more productive strategy that acknowledges the complex nature of the opioid crisis.

## Data Availability

The data analyzed in this study is subject to the following licenses/restrictions: this data is available through a signed data use agreement with the Centers for Medicare & Medicaid Services. Requests to access these datasets should be directed to https://www2.ccwdata.org/web/guest/home/.
